# Artificial Intelligence (AI) and Parathyroid Gland Preservation During Thyroidectomy

**DOI:** 10.7759/cureus.113496

**Published:** 2026-07-28

**Authors:** Antreana Hadjipavlou, Antria Nicolaou, Eleftheria Sazou, Ioannis Pliakos, Moysis Moysidis, Theodossis Papavramidis, Angeliki Chorti

**Affiliations:** 1 School of Medicine, Aristotle University of Thessaloniki, Thessaloniki, GRC; 2 1st Propaedeutic Department of Surgery, American Hellenic Educational Progressive Association (AHEPA) University Hospital, Aristotle University, Thessaloniki, GRC; 3 Department of Minimally Invasive Endocrine Surgery, Blue Cross Clinic, Thessaloniki, GRC

**Keywords:** artificial intelligence, near infrared autofluorescence, parathyroid gland, parathyroid gland preservation, thyroidectomy

## Abstract

Preserving the parathyroid glands (PGs) is crucial during thyroidectomy to avoid hypoparathyroidism and its related complications. Recently, artificial intelligence (AI) based techniques have been introduced intraoperatively, allowing surgeons to reduce the level of subjectivity and assist with the identification of PGs accurately and quickly. The aim of this narrative review is to assess the current evidence on the application of AI in parathyroid gland (PG) preservation. PubMed, Google Scholar, and Embase were used for the search for various types of studies and reviews dating from 2015 up to 2025, relating PG detection and preservation during thyroidectomy with AI. A total of 368 articles were identified through the database searching. After duplicates were removed and articles were screened, 358 records not fulfilling the criteria were excluded. In total, 11 studies are included, which focus on artificial intelligence or deep learning identification of parathyroid glands during thyroidectomy. The criteria of inclusion were the use or the development and training of an AI model, deep learning, near-infrared autofluorescence (NIRAF), and intraoperative PG identification and preservation. Although available data remain limited, this narrative review suggests that AI-assisted techniques are feasible and have the potential to improve surgical accuracy and efficiency. To confirm this clinical benefit, further high-quality prospective studies are required.

## Introduction and background

Thyroidectomy is one of the most frequently performed endocrine operations worldwide [1]; however, postoperative hypoparathyroidism remains one of its most common and clinically relevant complications [2]. To avoid postoperative hypocalcemia, an accurate assessment of PG vascularity is important during thyroidectomy [3]. PG damage, devascularization, autotransplantation, and/or unintentional resection can lead to postoperative transient or permanent hypocalcemia. Postsurgical hypoparathyroidism can detrimentally affect quality of life as symptoms can vary from neuromuscular irritability to cardiac arrhythmias and possible calcium disturbances [4]. It is a complication that can lead to severe symptoms, increased hospitalization, and even be life-threatening, requiring substitutive treatment and prolonged surveillance after discharge [5]. Therefore, during thyroid surgery, the PGs must be identified early and preserved [2].

Despite improvements in surgical techniques, the standard for preserving PGs is visual inspection, mainly depending on the operating surgeon’s experience. This subjective method has severe limitations due to the glands’ small size, variable location, and anatomical variations. Other techniques, however, may increase the surgical procedure's duration and expense [4]. A technique that assists with the identification of PGs accurately and quickly would be a beneficial intraoperative addition [6].

In recent years, AI has emerged as a promising field in surgical practices. Currently, AI applications in surgery, instead of being completely autonomous, are mostly assistive [7]. They have a great impact on surgical workflow efficiency, decision-making support, tissue recognition, image analysis, and prediction of patient outcomes [8]. AI encompasses a wide range of approaches, including machine learning (ML), deep learning (DL), computer vision (CV), and natural language processing (NLP), which have shown encouraging results across multiple surgical specialties. In thyroid surgery, the use of these technologies, especially CV, DL, and ML, is frequently in combination with optical imaging modalities like near-infrared autofluorescence (NIRAF), Indocyanine green (ICG) angiography, white-light video, and Laser Speckle Contrast Imaging (LSCI). They allow for technical PG detection and segmentation on intraoperative video, visual ischemia classification (PTAIR), and ex vivo tissue confirmation [9,10].

This narrative review aims to evaluate the current evidence on the use of AI in parathyroid gland preservation during thyroidectomy, focusing on the intraoperative performance using different surgical techniques.

## Review

Materials and methods

In this review, the databases PubMed, Google Scholar, and Embase were used for the manual searching of various studies in relation to AI and the identification and preservation of PGs during thyroidectomy. The following keywords were used in multiple combinations: AI, Artificial Intelligence, “Artificial Intelligence’[Mesh], "Machine Learning"[Mesh], machine learning*, deep learning*, "Parathyroid Glands"[Mesh], "Hypoparathyroidism"[Mesh], parathyroid*, parathyroid gland*, "Thyroidectomy"[Mesh] and gland preservation. In addition, we searched the reference lists of the identified articles for additional literature published on this topic. The timeframe used for the search was ten years, from January 2015 up to December 2025. All the studies selected are written in English, despite the country of origin being an English non-speaking country.

For the articles to be included in this narrative review, the following criteria had to be included the use of an AI model or any other form of DL, the use of NIRAF, the patients had completed a thyroid operation, the appropriate detection of PGs with sensitivity ≥90%, specificity ≥85%, or area under the curve (AUC) ≥0.90 and preservation of PGs, which is defined as preserved postoperative function with temporary hypocalcemia <20% and permanent hypocalcemia <3%. The grounds of exclusion include information not being related to the main objective of the review, studies with outcomes unrelated to parathyroid preservation (for example, only recurrent laryngeal nerve identification), and cases with distorted anatomy. In each of the studies qualified to be included, the evidence collected contains: the type of study, the study design, the types and numbers of databases used, the AI model used or developed, the name of the model or models, the number of patients used, the country it was contacted from if it was available, the type of the surgery the patients were undergoing, the number of the frames/images/videos used to train and validate the AI models, the comparison groups, the results of each study, the clinical relevance, the conclusions and the limitations if present.

Literature review

**Figure 1:**
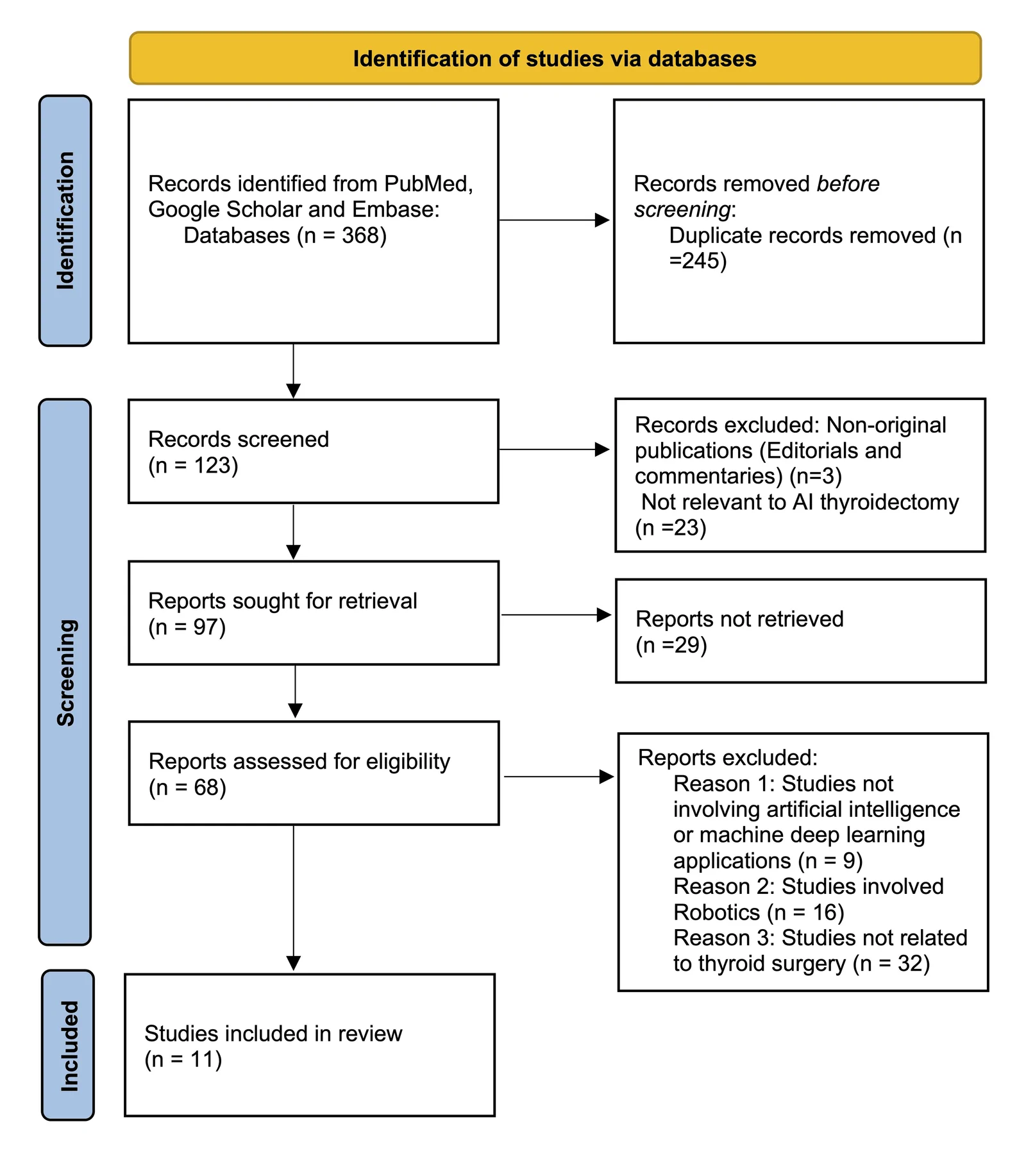
PRISMA flow diagram. PRISMA, Preferred Reporting Items for Systematic Reviews and Meta-Analyses.

A total of 368 records were identified through the search of the databases. After removal of duplicates, 50 articles were rejected, and the remaining 123 articles were screened. From these 123 records, three additional records were rejected because they were non-original publications, since they included editorials and commentaries. An additional 23 articles were rejected since they were not relevant to the use of AI during thyroidectomy. In total, 97 records were sought for retrieval, of which 29 were not retrieved. Then, from the 68 articles that were assessed for their eligibility, 57 were rejected. From these 57 records rejected, nine articles did not involve any artificial intelligence or the use of a deep learning model. In addition, 16 records were rejected since they included robotics, and 32 were also rejected because they did not have any relation to thyroid surgery. In total, 11 full-text articles were included in this review that include primary AI evidence for parathyroid identification (Figure 1).

The remaining references were used for supplementary information, hence the 26 references. The types of studies included are as follows: one randomized controlled trial (RCT), one prospective pilot, one evidence-based clinical guidelines study, one non-randomized comparative study, four systematic reviews and meta-analyses, six narrative reviews, six prospective cohorts, and six retrospective studies. The majority of the articles originated from China and the United States, with eight (31%) and five (19%) studies, respectively. In addition, six studies have been conducted in France and South Korea, consisting of three each, resulting in 11.5%. Only two studies (8%) have been conducted in Scandinavian areas: one in the Netherlands and the other was multinational, with the majority in Sweden. Moreover, the countries with only one study (4%) include Belgium, Canada, Italy, and the UK. Only one study was multinational (Table 1).

**Table 1:** Summary of included studies. RLN, recurrent laryngeal nerve; LSCI, Laser Speckle Contrast Imaging; NIRAF, near-infrared autofluorescence; MB, methylene blue; ICG, Indocyanine green; AI, artificial intelligence; OR, operating room; PTAIR, Parathyroid gland Artificial Intelligence Recognition; RGB, red-green-blue; PTH, parathyroid hormone; U-HRNet, U-shaped High-Resolution Network; DL, deep learning; NIR, near-infrared.

Author (year)	Country	Type of study	Technique/technology	Key findings
Bergenfelz et al. (2008) [1]	Scandinavia (multicenter)	Prospective cohort	Registry database (clinical follow-up and laryngoscopy)	RLN palsy 3.9%, permanent RLN palsy 0.97%, hypocalcemia 9.9% early and 4.4% at 6 months, re-bleeding 2.1%, infection 1.6%
Van Slycke et al. (2021) [2]	Belgium	Prospective cohort	Autofluorescence	35.0% vs 14.1% identification of all 4 glands; temporary hypoparathyroidism 17.5% vs 31.9%; permanent hypocalcemia 0% vs 3.1%
Mannoh et al. (2021) [3]	USA	Prospective cohort	LSCI	≥1 well-perfused gland predicted preserved function; 0% permanent hypoparathyroidism when a viable gland remained
Khan et al. (2022) [4]	International	Guidelines	None	Defines diagnosis and management of chronic hypoparathyroidism
Benmiloud et al. (2020) [5]	France	Randomized controlled trial	NIRAF	Hypocalcemia 9.1% vs 21.7%; accidental excision 3.3% vs 14.7%
Wong et al. (2020) [6]	Canada	Narrative review	MB, NIRAF, ICG imaging	Improvement in gland recognition with certain limitations; NIRAF sensitivity 80-100%
Gumbs et al. (2021) [7]	Italy	Narrative review	AI in OR	A step towards semi-autonomous intraoperative decisions
Birkhoff et al. (2021) [8]	Netherlands	Systematic review	AI in OR	Broad surgical use (80-95% accuracy)
Wang et al. (2024) [9]	China	Retrospective	PTAIR 2.0	Real-time surgical alerts enabled by early ischemia prediction (90-94% accuracy; AUC 0.9%)
Akgun et al. (2024) [10]	USA	Prospective cohort	AI and NIRAF	Increased identification accuracy (96.5% precision; 96.5% recall; AUC 0.985)
Liu et al. (2025) [11]	China	Prospective cohort	AI segmentation	High precision segmentation (70.7%-77.5% dice coefficient, greatest improvement medical students 66.2%-78.9%)
Kim et al. (2022) [12]	South Korea	Prospective cohort	AI-assisted dual-modality imaging (RGB and NIRAF)	Improvement of contrast and gland identification (94.5% precision)
Oh et al. (2022) [13]	South Korea	Prospective pilot	Handheld imaging	Reliable real-time identification (94.7% precision)
Wang et al. (2022) [14]	China	Retrospective	PTAIR	High recognition accuracy (88.7% precision; 87.5% recall rate)
Wang et al. (2022) [15]	China	Retrospective	PTAIR	Real-time AI assistance during surgery (96.9% recognition rate, 3.8 seconds earlier parathyroid gland recognition than senior surgeons)
Lin et al. (2023) [16]	China	Retrospective	PTAIR 2.0	High location and ischemia prediction (94.1% parathyroid gland early prediction; 98.9% identification; 92.1% ischemia alert)
Lee et al. (2024) [17]	South Korea	Retrospective	AI-driven RGB video detection confirmed by PTH washout	Improved detection accuracy (77% baseline precision; 79% advanced precision; 78.6% image inpainting model)
Sang et al. (2024) [18]	China	Retrospective	Video-U-HRNet (DL model)	Real-time segmentation during surgery videos (89.5% precision; 77.3% recall; 70.8% accuracy)
Thomas et al. (2019) [19]	USA	Non-randomized comparative	NIRAF	High identification accuracy (NIRAF post hoc analysis 81.8% sensitivity; 73.7% specificity; 78.8% accuracy)
Abbaci et al. (2018) [20]	France	Systematic review	Optical technologies	Improvement of gland identification
Wang et al. (2021) [21]	China	Meta-analysis	NIRAF	High sensitivity and specificity in gland identification (96% sensitivity; 96% specificity; AUC 0.99)
Yuan et al. (2023) [22]	China	Narrative review	NIRAF	Improved gland visualization (NIRAF 82% sensitivity, ICG 81% sensitivity)
Duh & Davis (2022) [23]	USA	Narrative review	AI and NIRAF	Future use of AI in surgery (90.5% sensitivity; 95.7% precision)
Korman & Patel (2025) [24]	USA	Narrative review	NIR and ICG	High AI contribution to gland identification (50-95% recall; 72-94% precision)
Falco et al. (2016) [25]	France	Narrative review	NIRAF	NIRAF indicates high reliability in gland identification (100% detection (n=28); fluorescence intensity: PG 40.6±26.5 vs thyroid 31.8±22.3 vs background 16.6±15.4 (p<0.0014); no postoperative hypocalcemia)
Edafe et al. (2014) [26]	UK	Systematic review and meta-analysis	Risk factor analysis	Transient hypocalcemia 27%, permanent 1%; female sex OR 2.28, autotransplantation OR 2.03, inadvertent parathyroid excision OR 1.90, Graves' disease OR 1.75

The 11 articles involving AI models [7-12,14-18] from 2022 show a clear predominance of the convolutional neural network (CNN)-based classification models, commonly used with the PTAIR family, and the DL segmentation (U-Net-based segmentation) models, which were commonly applied to white-light video or NIRAF imaging depending on the study. While two out of the 10 articles [7,8] discuss the use of AI in surgery, they did not train any models. The remaining nine articles [9-12,14-18] use and develop modern AI, machine learning, and deep models. The PTAIR model was developed by Wang et al. [14], who later applied it in surgery videos to check the eligibility of the PTAIR system by an early visual intraoperative demonstration [15]. Both articles use a PTAIR model in combination with the CNN. The first study by Wang et al. [14] validated the CNN system, while the second study [15] demonstrated gland identification in surgery videos using the same CNN approach. Later, the PTAIR 2.0, again CNN-based, was developed, which expanded its use from simple gland detection to a multitasking function of predicting the parathyroid gland location, improving the intraoperative identification, and assessing for the possibility of ischemia [16]. It was then that PTAIR 2.0 was validated in real surgical cases, proving its clinical application [9]. Therefore, the development and improvement of the PTAIR model is described in four articles [9,14-16], which focus on object detection from Faster R-CNN to YOLOX architectures. Similarly, Kim et al. and Liu et al. both address object detection using YOLOv5 and YOLOv11 [11,12]. Sang et al. use Video-U-HRNet for semantic segmentation [18]. Akgun et al. use 1-D NIRAF intensity data instead of images for signal classification [10]. Finally, Lee et al. focus on augmenting data applied to the object detection backbone [17]. In summary, 35% of the articles developed an AI model or a deep learning model.

The PTAIR family, Wang et al., and Lin et al. trained the model through white light endoscopic thyroid surgery images and videos, which were labelled by surgeons distinguishing parathyroid and non-parathyroid tissue. In addition, validation was performed through a single-center internal split dataset delineating sensitivity, specificity, and AUC with no external or multi-center validation [9,14-16]. The CNN-based image classification includes Kim et al., in which training of the model included labelled in situ and ex-vivo red-green-blue (RGB) and near-infrared images and an internal validation test set depicting sensitivity, specificity, and AUC [12]. In addition, Lee et al. trained the CNN model on intraoperative images from which data augmentation was achieved and internally validated the model by comparing the datasets with and without augmentation [17]. Akgun et al. developed a supervised AI classifier using ex vivo NIRAF signals with the ability to distinguish parathyroid and non-parathyroid tissue, analysing the accuracy of diagnosis to prospectively validate it [10]. Liu et al. developed their model by applying expert-labelled images obtained from endoscopic and robotic surgeries, validating it prospectively by contrasting different surgical approaches [11]. Sang et al. trained a video-based DL model through intraoperative thyroid surgery videos and retrospectively validated it by assessing frame-by-frame validity between predicted and surgeons' annotated PG sites [18]. In summary, no study in the included AI parathyroid identification literature provided external validation from multiple centers.

Several studies have demonstrated that the use of AI DL models possesses the ability to intraoperatively identify and segment PGs. Wang et al. and Lin et al. reported that the deep-learning models PTAIR and PTAIR 2.0 have the ability to identify parathyroid glands intraoperatively, to distinguish parathyroid tissue from adjacent structures, and to describe intraoperative ischemia in the setting of thyroidectomy [9,15,16]. Kim et al. demonstrated that a coaxial excitation, dual-RGB and near-infrared NIR paired imaging system is a feasible and effective way for intraoperative PG detection during thyroidectomy. This paired imaging approach improves tissue contrast to support localization of parathyroid glands intraoperatively [12]. Prospective studies by Akgun et al. and Liu et al. confirm the models’ performance in real-life surgical case settings, reporting adaptation, intraoperative identification of parathyroid glands using the assistance of AI, and separation of tissues in the endoscopic or robotic surgical approaches using different models and approaches [10,11]. In addition, retrospective studies such as Lee et al. and Sang et al. report the use of inpainting imaging and frame-to-frame detection methods for intraoperative gland visualization, along with describing the potential applications assisting and supporting during surgeries [17,18]. These findings suggest that AI-based methods may support intraoperative parathyroid gland identification and have potential use in surgical training and decision-making when compared with traditional methods.

Discussion

AI has emerged recently and has already shown promising results for PG identification during total thyroidectomy. It is supported by many studies that AI systems help to distinguish between thyroid tissue and parathyroid tissue, beyond what the naked eye can achieve. Correctly identifying the PG prevents any accidental damage or disruption to its blood supply, which would lead to postoperative hypoparathyroidism [20]. According to Edafe et al., an analysis of 115 observational studies found that the median (IQR) incidence of transient hypocalcemia was 27%, whereas the median incidence of permanent hypocalcemia was 1% [26].

Akgun et al. used 1151 ex vivo autofluorescence images from 678 surgical operations, including surgically removed parathyroid glands, thyroid glands, lymph nodes, and thymic tissue [10]. Two training models were employed: the first model included all specimen types, and the second model excluded thyroid to mitigate size bias. The AI models were based on DL, trained to detect autofluorescence signals specific to parathyroid glands. The proposed system demonstrated excellent diagnostic performance, with a precision and recall of 96.5% and an area under the curve of 0.985. False negative results were primarily observed in larger PGs or those with lower autofluorescence intensity [10].

Subsequent studies by Wang et al. [14,15] and Lin et al. [16] collectively represent the largest body of evidence evaluating PTAIR. PTAIR v1, developed by Wang et al., utilized white-light RGB endoscopic video and a Faster R-CNN DL algorithm to automatically identify PGs during endoscopic thyroid surgery [15]. In the initial study, the model achieved a recognition rate of 96.9% and identified PGs 3.8 seconds earlier than surgeons [15]. The data comprised 1700 annotated PG images extracted from 166 endoscopic thyroidectomy videos, with additional validation using 20 full-length videos. Comparison of several algorithms, including YOLOv3, Faster R-CNN, and Cascade R-CNN, demonstrated superior performance of Faster R-CNN, achieving precision, recall, and F1 scores of 88.7%, 92.3& and 90.5%, respectively [14,15].

Building upon this work, PTAIR 2.0 employed a YOLOX-based architecture to improve real-time intraoperative PG recognition from white-light endoscopic video. Lin et al. reported prediction, identification, and ischemia assessment accuracies of 94.1%, 98.9%, and 92.1%, respectively [16]. These findings suggest that PTAIR may serve as a useful decision-support tool for intraoperative PG identification and surgical training. However, no PTAIR study has yet evaluated downstream clinical outcomes, such as postoperative hypoparathyroidism, hypocalcemia or inadvertent parathyroid resection rates. Consequently, while the technology demonstrates promising diagnostic performance, its impact on long-term patient outcomes remains to be established.

In the study by Kim et al., a new method combined YOLOv5 architecture-based DL with a co-registered dual-RGB/NIR paired imaging device [12]. A coaxial excitation light of 785nm was introduced, along with a dual-sensor camera capturing both visible RGB images and NIR images at the same time, allowing the formation of a uniform illumination. The prototype was tested in situ and ex vivo on 10 patients, with different autofluorescence signals depending on the specimen, surgery type, and disease. The accuracy of the system was confirmed through resected specimens sent for analysis. The dual-RGB/NIR system conveys a stronger ability and performs better compared to the solitary use of NIR or RGB. It has achieved a precision of 94.7% with 19.5 millisecond inference time, supporting its suitability for intraoperative use [12].

In a separate study conducted in Sichuan Cancer Hospital in China, 210 intraoperative videos were obtained from robotic and endoscopic procedures [11]. Annotated video frames enabled models to localize and segment PG tissue. Out of the five models that were trained, YOLOv11 demonstrated superior performance for real-time PG detection. The final system, designated SmartThyroid, exhibited strong spatial overlap between predicted and reference PG segmentation, along with a more accurate performance and higher detection reliability [11]. In comparisons with surgeons, SmartThyroid exceeded the performance of junior surgeons and had a comparable performance to senior surgeons. Most importantly, the AI system improved the initial recognition rate of PG among surgeons, regardless of their level of expertise. This system shows promising results for future implementation into clinical settings [11].

The model Video-U-HRNet was developed to segment PG tissue in video frames. Its performance was evaluated against three other DL models, Video-TransUnet, Video-SwinUnet, and TransUnet, in addition to four surgeons, two being junior and the other two being senior [18]. The highest performance was reported by the Video-U-HRNet model, outperforming all the other AI models and surgeons of all expertise levels. An external cohort test was carried out, providing the following results: precision 89.5%, recall 77.3%, and accuracy 70.8%. Approximately 23% of parathyroid glands are missed by this system, and it demonstrates limited accuracy. Additionally, the surgeon comparator group is too small, making comparison challenging. Although the concept appears promising in theory, several limitations remain that warrant further refinement [18].

Lee et al. studied three datasets: baseline, geometric transformation, and generative network-based imaging inpainting. Out of the three, inpainting was determined to be the best way to detect PG tissue with the integration of an AI model, with confirmation by parathyroid (PTH) washout [17].

Despite the encouraging performance of AI, several limitations temper the momentum of these systems. Methodological constraints, leading to a lack of external validation, were the main issue for many studies, as they were conducted in single centers [25]. This limits patient variability, leading to a smaller range of different PG anatomies; therefore, it may not be suitable to apply these results on a larger scale. The reliance on small-center studies introduces potential operator-dependent variability and a lack of heterogeneity in age, comorbidities, and disease activity. Glandular histopathology can vary widely across different diseases, limiting the reliability of AI in accurately identifying PG and may even lead to the overestimation of the system’s accuracy. This limits the generalisability of the findings to real-world surgical settings [16]. Increasing the patient sample size may mitigate this unreliability. These limitations prevent the studies from being confidently translated to other centers, requiring further research with prospective validation in large-scale population-based studies before clinical adoption. Since a proportion of evidence is derived from a small number of influential studies, it could potentially skew perceptions of effectiveness [3,13]. No study in the included AI parathyroid identification literature provides external multicenter validation.

The following analysis has several limitations. Selection bias was introduced, as diagnostic definitions varied among studies and by restricting inclusion to English-language publications. The absence of simultaneously reported sensitivity and specificity limited the diagnostic accuracy assessment. Residual heterogeneity remained, despite subgroup analyses, while insufficient data prevented further stratified analyses [21].

The absence of histological confirmation of PG introduces the possibility of false-positive and false-negative results, hindering accurate calculation of sensitivity, specificity, and predictive values. This is a major concern, representing a fundamental weakness across many studies, as model performance is often evaluated against surgeon assessments rather than definitive tissue diagnoses [23].

In the study by Lee et al., one limitation is the annotation process, as it relies on the expertise of the person labelling the keyframes. It introduces subjective bias and may increase labour time [17]. Efforts to standardize annotation protocols could improve reproducibility [11,17].

Korman et al. conducted this systematic, comprehensive literature search and aimed to determine the applications of AI for augmented parathyroid gland recognition, with the use of eight studies [24]. Out of the eight studies, five emphasized the utility of AI intraoperatively for the identification of PG from surrounding tissues, and the remaining three studies focused on using AI to predict abnormal PGs from normal PGs. Several AI-based systems, including PTAIR, PTAIR 2.0, NIRAF, ICG, and video-based DL platforms, demonstrated high precision and recall rates that were often comparable to those of experienced endocrine surgeons and consistently superior to those of junior surgeons. However, most studies were conducted at single institutions using relatively small patient cohorts, raising concerns regarding selection bias and limited generalisability. The main factor that prevents postoperative hypoparathyroidism during parathyroidectomy is excellent recognition and identification of PG, to minimize the chance of any accidental resection. The studies used in this research support that AI models are promising with regard to the correct identification of PG, with eventual implementation of these technologies in large centers [24].

Furthermore, some studies may be affected by data leakage, particularly when the same patient’s results are used in both training and validation datasets, potentially inflating reported performance metrics. Finally, many studies do not adhere to AI-specific reporting frameworks, such as CLAIM (Checklist for Artificial Intelligence in Medical Imaging), TRIPOD-AI (Transparent Reporting of a Multivariable Prediction Model for Individual Prognosis or Diagnosis-Artificial Intelligence), and DECIDE-AI (Developmental and Exploratory Clinical Investigation of Decision-support Systems Driven by Artificial Intelligence), reducing transparency, reproducibility, and the ability to critically appraise methodological quality [27-29].

Implications and Future Directions

All these studies highlight the importance of correctly identifying the PG during total thyroidectomy, as it is very common during surgery to disrupt its blood supply or even remove it and damage it. These innovations aimed to complement the surgeons’ visual estimation and to decrease post-operative complications. AI models were found to outperform human visual ability in estimating and identifying the PG. Although the findings are encouraging, additional studies involving diverse patient populations across multiple centers are essential to establish the safety, efficacy, and practicality of this clinical implementation in the long term. Having a larger patient sample size ensures the results are generalisable and applicable to the wider population. Additionally, integrating AI with established imaging modalities and exploring real-time intraoperative guidance may further optimize outcomes. Lowering the rates of post-operative hypoparathyroidism as much as possible after total thyroidectomy can eventually increase quality of life, while minimizing the chance for prolonged hospitalization and substitutive treatment.

## Conclusions

The application of AI in addressing medical problems has been a subject of discussion for a long time now. Current literature has demonstrated that an intraoperative AI system could assist in the identification of PG with superior performance in technical tasks when compared to its non-AI alternatives. AI-assisted techniques in the OR are increasingly feasible and may reduce the risk of inadvertent gland injury, potentially reducing postoperative hypocalcemia. These techniques appear to function best as an addition to surgical expertise, in which AI complements rather than replaces human skills. However, while early studies show the feasibility of AI-assisted surgeries, more clinical research is needed to show its associated changes in current health systems, since all metrics are technical performance measures on retrospective data, with the included literature lacking clinical outcomes. In addition, no external multicenter validation exists for in-situ AI parathyroid detection in the included studies. Therefore, prospective multicenter trials with histologically confirmed inadvertent resection rates and standardized hypoparathyroidism endpoints are the necessary next step before clinical adoption.
